# Supplementary material for: Pathophysiological Significance of Neutrophilic Transfer RNA-Derived Small RNAs in Asymptomatic Moyamoya Disease
Source: Cells. 2021 May 1;10(5):1086. doi: 10.3390/cells10051086 (PMC8147334; doi:10.3390/cells10051086)

2D Structure

```
851                                     876
                                  8mer-1a
5'-ttGAACGTTTACCATT---CCATGTGGGa-3' utr
      |||   :| |||  :|    |||||||||
3'-ucCUUAGGAUUGGCGAUCUGGUACACCCu-5' tRF
                 16151413    7 6 5 4 3 2
            3' Pairing          Seed
```

Local AU

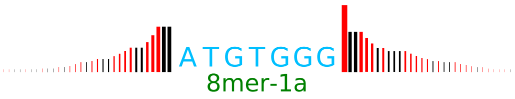

Position

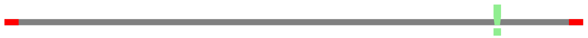

Supplement: Supplementary file 1 [file cells-10-01086-s001.zip › cells-1149161 3rd supplementary materials/Data Supplements/Data Supplement V.zip!/Data supplement 6-mRNAs targets of tsRNAs/tsRNA-Glu-TTC-010/Diagrams/tRF-Glu-TTC-010_vs_ENST00000421688(TMEM235).pdf]
